# HIV Treatment as Prevention: Systematic Comparison of Mathematical Models of the Potential Impact of Antiretroviral Therapy on HIV Incidence in South Africa

**DOI:** 10.1371/journal.pmed.1001245

**Published:** 2012-07-10

**Authors:** Jeffrey W. Eaton, Leigh F. Johnson, Joshua A. Salomon, Till Bärnighausen, Eran Bendavid, Anna Bershteyn, David E. Bloom, Valentina Cambiano, Christophe Fraser, Jan A. C. Hontelez, Salal Humair, Daniel J. Klein, Elisa F. Long, Andrew N. Phillips, Carel Pretorius, John Stover, Edward A. Wenger, Brian G. Williams, Timothy B. Hallett

**Affiliations:** 1 Department of Infectious Disease Epidemiology, Imperial College London, London, United Kingdom; 2 Centre for Infectious Disease Epidemiology and Research, University of Cape Town, Cape Town, South Africa; 3 Harvard School of Public Health, Boston, Massachusetts, United States of America; 4 Africa Centre for Health and Population Studies, University of KwaZulu-Natal, Mtubatuba, South Africa; 5 Department of Medicine, Stanford University, Stanford, California, United States of America; 6 Intellectual Ventures Laboratory, Bellevue, Washington, United States of America; 7 Research Department of Infection & Population Health, University College London, London, United Kingdom; 8 Medical Research Council Centre for Outbreak Analysis and Modelling, Department of Infectious Disease Epidemiology, Imperial College London, London, United Kingdom; 9 Erasmus University, Rotterdam, Netherlands; 10 Department of Primary and Community Care, Radboud University Nijmegen Medical Center, Nijmegen, Netherlands; 11 School of Science and Engineering, Lahore University of Management Sciences, Lahore, Pakistan; 12 Yale University, New Haven, Connecticut, United States of America; 13 Futures Institute, Glastonbury, Connecticut, United States of America; 14 South African Centre for Epidemiological Modelling and Analysis, Stellenbosch, South Africa; Duke University Medical Center, United States of America

## Abstract

**Background:**

Many mathematical models have investigated the impact of expanding access to antiretroviral therapy (ART) on new HIV infections. Comparing results and conclusions across models is challenging because models have addressed slightly different questions and have reported different outcome metrics. This study compares the predictions of several mathematical models simulating the same ART intervention programmes to determine the extent to which models agree about the epidemiological impact of expanded ART.

**Methods and Findings:**

Twelve independent mathematical models evaluated a set of standardised ART intervention scenarios in South Africa and reported a common set of outputs. Intervention scenarios systematically varied the CD4 count threshold for treatment eligibility, access to treatment, and programme retention. For a scenario in which 80% of HIV-infected individuals start treatment on average 1 y after their CD4 count drops below 350 cells/µl and 85% remain on treatment after 3 y, the models projected that HIV incidence would be 35% to 54% lower 8 y after the introduction of ART, compared to a counterfactual scenario in which there is no ART. More variation existed in the estimated long-term (38 y) reductions in incidence. The impact of optimistic interventions including immediate ART initiation varied widely across models, maintaining substantial uncertainty about the theoretical prospect for elimination of HIV from the population using ART alone over the next four decades. The number of person-years of ART per infection averted over 8 y ranged between 5.8 and 18.7. Considering the actual scale-up of ART in South Africa, seven models estimated that current HIV incidence is 17% to 32% lower than it would have been in the absence of ART. Differences between model assumptions about CD4 decline and HIV transmissibility over the course of infection explained only a modest amount of the variation in model results.

**Conclusions:**

Mathematical models evaluating the impact of ART vary substantially in structure, complexity, and parameter choices, but all suggest that ART, at high levels of access and with high adherence, has the potential to substantially reduce new HIV infections. There was broad agreement regarding the short-term epidemiologic impact of ambitious treatment scale-up, but more variation in longer term projections and in the efficiency with which treatment can reduce new infections. Differences between model predictions could not be explained by differences in model structure or parameterization that were hypothesized to affect intervention impact.

*Please see later in the article for the Editors' Summary*

## Editors' Summary

**Background:**

Following the first reported case of AIDS in 1981, the number of people infected with HIV, the virus that causes AIDS, increased rapidly. In recent years, the number of people becoming newly infected has declined slightly, but the virus continues to spread at unacceptably high levels. In 2010 alone, 2.7 million people became HIV-positive. HIV, which is usually transmitted through unprotected sex, destroys CD4 lymphocytes and other immune system cells, leaving infected individuals susceptible to other infections. Early in the AIDS epidemic, half of HIV-infected people died within eleven years of infection. Then, in 1996, antiretroviral therapy (ART) became available, and, for people living in affluent countries, HIV/AIDS gradually became considered a chronic condition. But because ART was expensive, for people living in developing countries HIV/AIDS remained a fatal condition. Roll-out of ART in developing countries first started in the early 2000s. In 2006, the international community set a target of achieving universal ART coverage by 2010. Although this target has still not been reached, by the end of 2010, 6.6 million of the estimated 15 million people in need of ART in developing countries were receiving ART.

**Why Was This Study Done?:**

Several studies suggest that ART, in addition to reducing illness and death among HIV-positive people, reduces HIV transmission. Consequently, there is interest in expanding the provision of ART as a strategy for reducing the spread of HIV (“HIV treatment as prevention"), particularly in sub-Saharan Africa, where one in 20 adults is HIV-positive. It is important to understand exactly how ART might contribute to averting HIV transmission. Several mathematical models that simulate HIV infection and disease progression have been developed to investigate the impact of expanding access to ART on the incidence of HIV (the number of new infections occurring in a population over a year). But, although all these models predict that increased ART coverage will have epidemiologic (population) benefits, they vary widely in their estimates of the magnitude of these benefits. In this study, the researchers systematically compare the predictions of 12 mathematical models of the HIV epidemic in South Africa, simulating the same ART intervention programs to determine the extent to which different models agree about the impact of expanded ART.

**What Did the Researchers Do and Find?:**

The researchers invited groups who had previously developed mathematical models of the epidemiological impact of expanded access to ART in South Africa to participate in a systematic comparison exercise in which their models were used to simulate ART scale-up scenarios in which the CD4 count threshold for treatment eligibility, access to treatment, and retention on treatment were systematically varied. To exclude variation resulting from different model assumptions about the past and current ART program, it was assumed that ART is introduced into the population in the year 2012, with no treatment provision prior to this, and interventions were evaluated in comparison to an artificial counterfactual scenario in which no treatment is provided. A standard scenario based on the World Health Organization's recommended threshold for initiation of ART, although unrepresentative of current provision in South Africa, was used to compare the models. In this scenario, 80% of HIV-infected individuals received treatment, they started treatment on average a year after their CD4 count dropped below 350 cells per microliter of blood, and 85% remained on treatment after three years. The models predicted that, with a start point of 2012, the HIV incidence would be 35%–54% lower in 2020 and 32%–74% lower in 2050 compared to a counterfactual scenario where there was no ART. Estimates of the number of person-years of ART needed per infection averted (the efficiency with which ART reduced new infections) ranged from 6.3–18.7 and from 4.5–20.2 over the periods 2012–2020 and 2012–2050, respectively. Finally, estimates of the impact of ambitious interventions (for example, immediate treatment of all HIV-positive individuals) varied widely across the models.

**What Do These Findings Mean?:**

Although the mathematical models used in this study had different characteristics, all 12 predict that ART, at high levels of access and adherence, has the potential to reduce new HIV infections. However, although the models broadly agree about the short-term epidemiologic impact of treatment scale-up, their longer-term projections (including whether ART alone can eliminate HIV infection) and their estimates of the efficiency with which ART can reduce new infections vary widely. Importantly, it is possible that all these predictions will be wrong—all the models may have excluded some aspect of HIV transmission that will be found in the future to be crucial. Finally, these findings do not aim to indicate which specific ART interventions should be used to reduce the incidence of HIV. Rather, by comparing the models that are being used to investigate the feasibility of “HIV treatment as prevention," these findings should help modelers and policy-makers think critically about how the assumptions underlying these models affect the models' predictions.

**Additional Information:**

Please access these websites via the online version of this summary at http://dx.doi.org/10.1371/journal.pmed.1001245.

- This study is part of the July 2012 *PLoS Medicine* Collection, Investigating the Impact of Treatment on New HIV Infections
- Information is available from the US National Institute of Allergy and Infectious Diseases on HIV infection and AIDS
- NAM/aidsmap provides basic information about HIV/AIDS and summaries of recent research findings on HIV care and treatment
- Information is available from Avert, an international AIDS charity on many aspects of HIV/AIDS, including information on HIV/AIDS treatment and care, on HIV treatment as prevention, and on HIV/AIDS in South Africa (in English and Spanish)
- The World Health Organization provides information about universal access to AIDS treatment (in English, French, and Spanish); its 2010 ART guidelines can be downloaded
- The HIV Modelling Consortium aims to improve scientific support for decision-making by coordinating mathematical modeling of the HIV epidemic
- Patient stories about living with HIV/AIDS are available through Avert; the charity website Healthtalkonline also provides personal stories about living with HIV, including stories about taking anti-HIV drugs and the challenges of anti-HIV drugs

## Introduction

There has recently been increasing interest in expanding provision of antiretroviral therapy (ART) as a tool for reducing the spread of HIV in generalised epidemics in sub-Saharan Africa [1]–[5]. As momentum gathers for “HIV treatment as prevention", there is an urgent need to understand how ART might contribute to averting HIV transmissions, in addition to its direct benefits in reducing morbidity and mortality amongst treated patients. Mathematical modelling has supplied critical insights to discussions about treatment as prevention by providing a framework for combining information about the relationship between an infected individual's viral load and HIV transmissibility [6],[7], the reduction in a host's HIV viral load when on ART [8],[9], and the population-level contact structure over which HIV is transmitted [10],[11].

The idea of using medicines that suppress viral concentrations to reduce transmission of infection was posed almost as soon as the first HIV drugs were developed [12],[13]. Early models of the impact of ART focused on the opposing effects of reduced transmissibility and extended survival on new HIV infections, and whether associated increases in sexual risk behaviour would negate the prevention benefits of ART [10],[12],[14]–[23]. Since then, longitudinal observational data and one randomized controlled trial have demonstrated substantial reductions in the risk of heterosexual HIV transmission when the infective partner is virally suppressed [24]–[28], and continued follow-up of individuals receiving ART has confirmed the durability of viral suppression [29], including in sub-Saharan Africa [30],[31]. At the same time, there have been tremendous improvements in access to treatment in sub-Saharan Africa [32]. More recent modelling has shown more optimism about the potential for treatment to reduce new HIV infections in this region, with much work focused on the setting of South Africa, home to one in six people living with HIV globally [33].

Perhaps the most provocative of these modelling efforts has been the study by Granich and colleagues suggesting that a strategy involving annual testing and immediate treatment for all HIV-infected individuals, combined with other interventions, could eliminate HIV by the year 2050 [34]. Wagner and Blower implemented the same model but used different assumptions about treatment uptake amongst asymptomatic infected individuals that they characterised as being more realistic, and concluded that elimination would not be possible [35]. Kretzschmar et al. highlighted how choices in model structure affect the epidemic dynamics and intervention impact [36]. Dodd et al. showed that the potential for treatment to eliminate HIV depends on the patterns of sexual mixing in the population [11]. An age-structured model by Bacaër et al. found that elimination might be possible with less frequent testing than proposed by Granich et al., given recent epidemic trends and increases in condom usage [37]. Bendavid et al. used a microsimulation model to highlight that, in addition to increasing HIV testing, improving linkage to and retention in care are essential to achieving maximal benefits of test-and-treat interventions [38].

Other models have focused on the potential prevention benefits of providing treatment in line with current therapeutic guidelines. Eaton et al. estimated that 60 to 90 new infections could be averted for every 1,000 additional persons treated with CD4 cell count below 350 cells/µl (the current World Health Organization recommendation for when to start treatment [39]), depending on how well patients on treatment are retained in care [40]. The Goals model, used in the evaluation of the new UNAIDS Investment Framework, found that a US$46.5 billion incremental investment over the years 2011 to 2020, incorporating expanding access to ART, could avert 12.2 million new infections and 7.4 million deaths globally over that period [41]. Using a microsimulation model of the HIV epidemic in KwaZulu-Natal Province, Hontelez et al. found that expanding access to ART from those with CD4 cell count ≤200 cells/µl to those with ≤350 cells/µl required 28% more patients to receive treatment, but amounted to only a 7% increase in annual investment [42]. Cumulative net costs broke even after 16 y.

Models have also sought to understand the impact of past and current treatment policies; Johnson et al. used the ASSA2003 and STI-HIV Interaction models to assess the relative contributions of increased condom usage and ART scale-up to the declines in HIV incidence in South Africa up to 2008 [43]. Finally, other mathematical models have been used for short-term projections as a basis for power calculations for community-randomized trials of treatment as prevention [44].

Each of these models has predicted dramatic epidemiologic benefits of expanding access to ART, but models appear to diverge in their estimates of the possibility of eventually eliminating HIV using ART, the cost-effectiveness of increasing the CD4 threshold for treatment eligibility, and the benefits of immediate treatment compared to treatment based on the current World Health Organization eligibility guidelines. Directly comparing the models' predictions is challenging because each model has been applied to a slightly different setting, has used different assumptions regarding other interventions, has been used to answer different questions, and has reported different outcome metrics.

In this study we seek to understand the extent to which diverse mathematical models agree on the epidemiological impact of expanded access to ART by simulating the same set of intervention scenarios across the models and focusing on standardised outputs. The intervention scenarios are designed to be simple enough to be consistently implemented across different types of models in order to control several aspects of the treatment programme and isolate the effects of model structure, parameters, and assumptions about the underlying epidemic on estimates of intervention impact. The purpose of this study is not to make predictions about the impact of any particular intervention in any specific setting, but rather to better characterise the array of mathematical models being used to inform policy about treatment as prevention in hyperendemic settings such as South Africa.

## Methods

### Study Design

Literature and reports of meetings on related topics were reviewed in August 2011, and researchers who had previously developed mathematical models of the potential epidemiological impact of expanded access to ART, calibrated to the South African epidemic setting, were invited to participate in the model comparison exercise by simulating a standardised set of ART scale-up scenarios. Three aspects of the treatment programme were systematically varied: the CD4 threshold for treatment eligibility, access to treatment for those eligible, and the retention of patients on treatment. The timing of ART introduction and the rate at which individuals start treatment after becoming eligible were also standardised. The impact of an intervention was measured by comparing the number of new infections in the intervention scenario with that in a counterfactual epidemic simulation in which no ART is provided within the same model population. The counterfactual of no ART was chosen so that comparison between models would be independent of assumptions about the historic growth in ART uptake. As such, the results should not be interpreted as estimates of the future impact of treatment compared to current patterns of ART coverage, but can be generally taken as estimates of the overall net impact of treatment in a hypothetical scenario that assumes rapid ART scale-up and a homogenous rate of ART initiation across all ART-eligible adults. Although different models may incidentally have been calibrated using the same data, no standardisation was imposed on the specific epidemiologic data used for model calibration or on the calibration procedure itself in this exercise.

### Mathematical Models

Twelve groups accepted the invitation to participate in the model comparison exercise. The collection of models encompasses a wide range of model structures, mechanisms for representing HIV transmission and disease progression, overall levels of complexity, and detail in the characterisation of treatment programmes. Table 1 summarises the names, authors, and key references for each model, and compares aspects of model structure. Four of the models are agent-based microsimulation models (i.e., models that track the behaviour and infection status of individual people) and use random-number generators to simulate particular events such as a new partnership formation or transmission events. The remaining eight models are deterministic compartmental models that stratify the population into groups according to each individual's characteristics and HIV infection status and use differential or difference equations to track the rate of movement of individuals between these groups. One of the models, the BBH model, solves the differential equations analytically, while the others numerically evaluate the differential equations. Ten of the models explicitly simulate both sexes and heterosexual HIV transmission, and six of the models include some form of age structure, although the extent to which age affects the natural history of HIV, the risk of HIV acquisition, and the risk of HIV transmission varies amongst these. All of the models simulate the national HIV epidemic in South Africa except for the STDSIM model, which simulates the higher prevalence Hlabisa subdistrict of KwaZulu-Natal Province, South Africa. Box 1 gives further comparative description of the structures and parameterization of the mathematical models.

**Table 1:** Description of mathematical models.

Model Name [Key References]	Model Authors	Model Type	Model Calibration	Population Structure	Sexual Mixing	Variation in Infectiousness over Course of Infection	Increased Male-to-Female Transmission?	Reduced Transmission on ART	Behaviour Change in 2000s
BBH	Till Bärnighausen, David Bloom, Salal Humair	Deterministic (analytically derived)	Initialized to epidemic state in year 2009; HIV incidence generated using national data on HIV prevalence, mortality, ART, and sexual behaviour	Two-sex, age 15–49 y	Homogeneous heterosexual mixing	Early infection, latent infection, late-stage/AIDS	3 times greater	96.5%	Not applicable
Bendavid [38]	Eran Bendavid	Microsimulation	Calibrated to current epidemic state in year 2012; select parameters estimated by scanning ranges from literature.	Two-sex, age-structured	Short- and long-term partners; heterogeneity in number of partners; decrease with age; short partners assortative by 5-y age group	Early infection, then according to VL (partner VL not tracked, randomly sampled from population)	No, but includes circumcision	91% (mediated by VL)	Not applicable
CD4 HIV/ART (CD4 Model of HIV and ART) [77]	John Stover, Carel Pretorius	Deterministic	Incidence curve imported from Spectrum model [78] projection for South Africa 2009 national estimates	Single-sex, age 15+ y	Sexual mixing not explicitly modelled; infection by multiplying fixed force of infection by current HIV prevalence	Early infection (×9.2), asymptomatic, symptomatic (×7.3)	Not applicable	96%	Not applicable (incidence curve input from Spectrum)
Eaton	Jeffrey Eaton, Timothy Hallett, Geoffrey Garnett	Deterministic	Sex-specific age (15–49 y) HH prevalence (HSRC '02, '05, '08) and ANC prevalence '90–'08 estimated using Bayesian framework	Two-sex, age 15–49 y (sexually active) and age 50+ y (not sexually active)	Three sexual risk groups, with partially assortative mixing by risk group	Early infection (×38), CD4>350 (×0.61), 350>CD4>200, 200>CD4>100 (×3.75), CD4≤100 (×0.71)	No	92%	Reduction in unprotected sexual contacts over ∼1999 to 2011 (timing and amount estimated)
EMOD	Daniel Klein, Anna Bershteyn, Edward Wenger, Karima Nigmatulina, Philip Eckhoff	Microsimulation	HIV prevalence time series (ANC '90–'09) and by age and sex (HSRC '08); sexual behaviour informed by Africa Centre for Health and Population Studies [79]	Two-sex, age-structured	Transitory, informal, and marital relationships; heterogeneity in propensity for each type of relationship by age and sex	Early infection (×26), asymptomatic, AIDS (×7.2)	No, but includes male circumcision	96%	Increase in condom usage; most change occurs between 1999 and 2009
Fraser	Christophe Fraser	Deterministic	UNAIDS age 15–49 y HIV prevalence estimates, fit by least squares	Two-sex, age 15–49 y	Three sexual risk groups, with partially assortative mixing by risk group	Early infection (×26, adjusted for partner duration), CD4>200, CD4≤200 (×2.4)	No, but 76% of males are circumcised (based on Western Cape)	90%	No
Goals [41],[80]	Carel Pretorius, John Stover	Deterministic	Calibrated to match time series in HIV prevalence from Spectrum projection	Two-sex, age 15–49 y	Not sexually active, low, medium, high (CSW and clients) risk, plus IDU and MSM; mixing perfectly assortative by risk group except low risk mix with CSW client	Early infection (×28), asymptomatic, symptomatic (×7.3)	1.4 times greater	92%	Increase in condom usage over 1996 to 2009
Granich [34]	Brian Williams, Reuben Granich	Deterministic	Calibrated to annual national age 15–49 y prevalence estimates	Single-sex, age 15–49 y	Homogeneous	No	Not applicable	99%	No
HIV Portfolio [81]	Elisa Long, Margaret Brandeau, Douglas Owens	Deterministic	Initialized to current epidemic state in year 2011	Two-sex, age 15–49 y	Homogeneous	Early infection (∼×5), CD4>350, 350>CD4>200 (∼×1.6), CD4≤200 (∼×2)	∼1.5 times greater, plus circumcision	90%	Not applicable
STDSIM [42],[82],[83]	Jan Hontelez, Sake de Vlas, Frank Tanser, Roel Bakker, Till Bärnighausen, Marie-Louise Newell, Rob Baltussen, Mark Lurie	Microsimulation	Calibrated to the Hlabisa subdistrict of KwaZulu-Natal using data from the Africa Centre for Health and Population Studies [79]	Two-sex, age-structured	Marriages, casual partnerships, and commercial sex; heterogeneity in propensity to form each type or relationship; changes with age	Early infection (×15), asymptomatic, symptomatic (×3), AIDS (×7.5)	2 times greater, plus circumcision	92%	Increase in condom usage and improvement in STI treatment over 1995 to 2003
STI-HIV Interaction [43],[84]	Leigh Johnson	Deterministic	Age-specific household prevalence (HSRC '02, '05, '08) and age-specific ANC prevalence ('90–'08) estimated using Bayesian framework	Two-sex, age-structured (5-y age groups)	High and low risk assortatively mixing; further stratified by short- and long-term partnerships, plus CSW	Early infection (×10), asymptomatic, pre-AIDS (×2.5), AIDS (×5)	∼2.5 times greater	90% (range: 78%–98%)	Increase in condom usage; most of increase occurs between 1995 and 2005
Synthesis Transmission [66]	Valentina Cambiano, Andrew Phillips, Deenan Pillay, Jens Lundgren, Geoff Garnett	Microsimulation	Calibrated using national HH survey data (HSRC '02, '05, '08)	Two-sex, age-structured	Primary partner and short-term partners; four different risk groups for propensity for short-term partners; semi-assortative by age	Early infection, then transmission determined by VL (primary partner's VL tracked, short-term sampled from population VL distribution)	∼1.5 times greater	Determined by adherence and viral load	Reduction in number of unprotected partners over period 1996 to 2008

ANC, antenatal clinic; CSW, commercial sex worker; HH, household; HSRC, South African Human Sciences Research Council; IDU, injecting drug user; MSM, men who have sex with men; STI, sexually transmitted infection; VL, viral load.

**Box 1. Comparative Description of Mathematical Models**

This box elaborates on the comparison of aspects of the models' structure, assumptions, and parameterization presented in Table 1. Specific details about the structure and implementation of each of the models are available in the references included in Table 1 or from the HIV Modelling Consortium (http://www.hivmodelling.org/plos-medicine-special-collection).

Many of the models allow individuals to have different propensities for sexual risk behaviour. Each of the microsimulation models allows individuals to have both long-term (or marital) partnerships and short-term (or informal or casual) partnerships that are different in duration, and individuals have heterogeneous propensities to form short-term partnerships. In the STDSIM model a proportion of the population engages in commercial sex work partnerships; in the EMOD model a proportion can have transitory partnerships, a third partnership type that is shorter than a casual partnership. Among the microsimulation models, EMOD and STDSIM explicitly simulate the sexual partnership network, while the Bendavid and Synthesis Transmission models calculate the risk of acquiring HIV for an individual in a partnership by sampling the distribution of viral load across, respectively, the entire population and potential partners.

The deterministic models assume that sexual contacts occur instantaneously. The BBH, Granich, and HIV Portfolio models assume that all individuals form new contacts at the same rate and mix homogeneously. The other deterministic models stratify the population into risk groups that form new contacts at different rates (Eaton, Fraser, and Goals: three groups; STI-HIV Interaction: two groups). The STI-HIV Interaction and Goals models additionally include commercial sex workers, and the Goals model includes transmission among men who have sex with men and injecting drug users. The STI-HIV Interaction model separates both the low- and high-risk groups into those with short-term or long-term partnerships or both. The Eaton, Fraser, and STI-HIV Interaction models all include a degree of “assortative" mixing (preferential partnership formation with those in the same risk group), and all partnerships are formed in the same risk group in the Goals model, except for low-risk men and women who are married to high-risk partners. The CD4 HIV/ART model does not explicitly model sexual mixing but rather calculates the number of new HIV infections by multiplying the current number of HIV-infected adults by a fixed force of infection calculated from the Spectrum model projection for South Africa.

All of the models except for the Granich model simulate different stages of HIV infection that affect the transmissibility of an individual, including a period of elevated infectiousness during the first few weeks of infection and increased transmission during later stage infection. Parameters governing the relative transmissibility during early infection are based principally on two sources: a meta-analysis of HIV transmission per coital act by Boily et al. [68], which estimated a 10-fold increase in transmission relative to asymptomatic infection (BBH, CD4 HIV/ART, Goals, and STI-HIV Interaction), or a reanalysis of data from Rakai, Uganda [70], by Hollingsworth et al. [69], which estimated a 26-fold increase (Eaton, EMOD, Fraser, and Synthesis Transmission). Relative transmissibility after the early stage is according to clinical stage (asymptomatic and AIDS: BBH, CD4 HIV/ART, EMOD, Goals; asymptomatic, pre-AIDS, and AIDS: STDSIM, STI-HIV Interaction) or CD4 count (Eaton, Fraser, and HIV Portfolio). The Bendavid and Synthesis Transmission models simulate the change in viral load for infected individuals and associate HIV transmission with this according to an empirically described relationship [6]. Many models assume an increased risk of male-to-female transmission compared to female-to-male transmission, and attenuation in female-to-male transmission due to male circumcision. The Goals, STDSIM, and Synthesis Transmission models include an increased risk of HIV transmission in the presence of other sexually transmitted infections.

The models that simulate each individual's viral load (Bendavid and Synthesis Transmission) mechanistically relate reduction in transmission on treatment to the effect of ART on viral load, while the other models all assume a reduction in transmission of greater than 90% for individuals on ART. The Bendavid, Eaton, and Synthesis Transmission models simulate a period of a few months of incomplete viral suppression after ART initiation before the full reduction in infectiousness is achieved. These three models and EMOD include a return to higher infectiousness during treatment failure. The remaining models assume a fixed reduction in transmissibility as soon as treatment is started, until either death on ART or dropout from treatment. The Bendavid and Synthesis Transmission models simulate switching to second-line ART upon an immunologic (Bendavid) or virologic (Synthesis Transmission) failure event. The Synthesis Transmission model is the only model to explicitly simulate heterogeneous adherence to treatment between patients and the emergence and impact of resistance. The models vary in their assumptions about what happens to an individual after dropping out from treatment. The CD4 HIV/ART, Fraser, Goals, Granich, and HIV Portfolio models return individuals who drop out to an untreated state, allowing them to restart treatment in exactly the same manner as those that have never been treated, while the Bendavid, STDSIM, STI-HIV Interaction, and Synthesis Transmission models do not allow individuals to start treatment again in the implementation for this exercise. Eaton allows individuals to restart treatment, but only once, and EMOD allows half of individuals to restart treatment after they once again satisfy the eligibility criterion.

Eleven of the models simulate the South African national HIV epidemic, while the STDSIM model has been calibrated specifically to the higher prevalence Hlabisa subdistrict of KwaZulu-Natal Province, South Africa. Nine models were calibrated to reproduce the historical time series of HIV prevalence in South Africa, while the BBH, HIV Portfolio, and Bendavid models were initialized using the current epidemic state in the years 2009, 2011, and 2012, respectively, and simulated forward from that point.

Most of the models were calibrated to yield a single set of model parameters and outputs. Two of the models (Eaton and STI-HIV Interaction) were calibrated using a Bayesian framework allowing for uncertainty in model parameters, which produces a joint posterior distribution of parameter combinations consistent with the observed HIV epidemic [43],[84]. The STI-HIV Interaction model allows for uncertainty in sexual behaviour, the natural history of HIV infection, and the effect of ART, while the Eaton model only allows for uncertainty in sexual behaviour and sexual mixing parameters.

Many of the models include facilities to simulate HIV testing and diagnosis, retention in care prior to treatment eligibility, and other processes related to achieving successful treatment, but these were not implemented for this exercise in order to conform to the simple intervention scenarios.

### Intervention Scenarios

Three different CD4 cell count thresholds for treatment eligibility were considered: CD4 count ≤200 cells/µl, CD4 count ≤350 cells/µl, and all HIV-infected individuals. In each eligibility scenario, treatment initiation was simulated under the assumption that all eligible individuals had equal access, without prioritisation for any subpopulations. It was further assumed that eligible individuals with access to the intervention would initiate ART at a constant rate after reaching eligibility, such that average time from eligibility to treatment initiation would be 1 y.

Treatment access was defined as the proportion of eligible individuals who eventually initiate treatment. For example, 60% access and eligibility at CD4≤350 cells/µl implies that 60% of individuals will initiate treatment, on average 1 y after their CD4 count drops below 350 cells/µl, while 40% will never access treatment. Seven levels of treatment access were evaluated: 50%, 60%, 70%, 80%, 90%, 95%, and 100%.

Retention was defined as the percentage of individuals remaining on treatment after 3 y, excluding from both the numerator and the denominator those who had died while on treatment. The levels of retention were 75%, 85%, 95%, and 100% (no dropout), with individuals dropping out from treatment at a constant rate such that the desired level of retention was achieved at the 3-y time point. The prognosis and future treatment options for individuals who dropped out from treatment were not standardised.

### Intervention Scale-Up

ART was assumed to be introduced into the population from the beginning of year 2012, with no treatment provision prior to this (in contrast to the rapid scale-up of treatment that has actually occurred prior to 2012 in South Africa). Intervention scale-up was immediate—a fraction (corresponding to the specified level of ART access) of individuals already eligible for treatment at the start of the intervention period were assumed to initiate treatment at a constant rate from that point, along with individuals who became eligible for treatment after the start of the intervention period.

### Output Metrics

The measures of intervention impact were the percentage reduction in HIV incidence rate among adults (aged 15–49 y) in the ART scenario versus the no-ART counterfactual, the cumulative number of person-years of ART provided since the introduction of ART, and the cumulative number of person-years of ART provided per infection averted as a measure of the “efficiency" with which ART prevents infections. The percentage reduction in incidence was defined by calculating the difference in the adult HIV incidence rate between the intervention and no-ART counterfactual in the same year and dividing this by the incidence rate in the counterfactual scenario. The number of person-years of ART provided per infection averted was calculated by dividing the cumulative number of person-years of ART by the difference between the number of new adult infections since year 2012 in the intervention and the counterfactual scenario. Each of these metrics was reported at the midpoints of the years 2020 and 2050. Most of the models included in this study were not designed with the intention of making realistic projections to year 2050, but these results were included to gain some insight into the long-term dynamics of the models.

In addition to these measures of intervention impact, each model reported the HIV prevalence and HIV incidence rate amongst males and females aged 15–49 y for the no-treatment counterfactual simulation and the total size of the adult population (age 15 y and older). Each model also produced the proportion of the HIV-infected population in each CD4 count category (≤200, 200–350, and >350 cells/µl) and in early HIV infection in year 2012, and the proportion of HIV transmissions from individuals in each category.

The Eaton and STI-HIV Interaction models report posterior means and 95% credible intervals for model outcomes of interest (see Box 1). The Bendavid model completed simulations only for 50%, 80%, and 100% access, and 75%, 85%, and 100% retention scenarios, and only simulated to year 2040, so results for this model are reported for the year 2040 where other model results are reported for year 2050. The BBH model completed simulations only for the 85% and 100% retention scenarios. The Granich model did not simulate ART for the CD4≤200 cells/µl eligibility threshold, while the STI-HIV Interaction model did not simulate ART eligibility for all HIV-infected individuals. As a result of these models not completing all intervention scenarios and outputs, some analyses include only a subset of the models. To maximise comparability, the 40% reduction in transmission due to combination with other preventive interventions assumed by Granich and colleagues in [34] was not incorporated here.

### Scenarios Representing the Existing ART Programme in South Africa

In a separate analysis, seven of the models (CD4 HIV/ART, Eaton, Fraser, Goals, Granich, STDSIM, and STI-HIV Interaction) were used to estimate the impact that the existing scale-up of ART in South Africa has had on HIV incidence and prevalence by comparing model simulations that include the ART scale-up over the past decade with the no-ART counterfactual. Models either used an existing calibration to the number of people on ART in South Africa (Fraser and STDSIM) or were calibrated using estimates of the number of adults starting and on ART in each year from 2001 to 2011 [45] (CD4 HIV/ART, Eaton, Goals, Granich, and STI-HIV Interaction).

Five models (Bendavid, CD4 HIV/ART, Eaton, Goals, and Granich) constructed short-term projections of HIV incidence in South Africa assuming different trajectories for continued ART scale-up from 2011 to 2016, the period covered by South Africa's national strategic plan [46]. Starting from the number of patients on ART in mid-2011, the numbers of adults starting ART in each of the years from mid-2011 through mid-2016 was specified. A “baseline" scenario was considered in which 400,000 adults would start ART in each of the next 5 y (approximately the number who started ART in 2009), for a total of 2 million new adults initiating ART. Three other scenarios were considered for the total numbers starting ART over the same period: (i) “low"—1.2 million start ART; (ii) “medium"—3 million start ART; and (iii) “high"—3.9 million start ART. (The exact number starting in each year is listed in Table 2.) The HIV incidence rate at the midpoint of 2016 and the cumulative number of new adult HIV infections over the period 2011 to 2016 were reported for each of these scenarios. For these projections, assumptions regarding CD4 distributions at ART initiation and rates of retention were based on actual treatment guidelines and programme experiences, but were not standardised across models.

**Table 2:** Number of adults starting ART each year in the short term.

Year	“Low" Future Scale-Up	“Baseline" Future Scale-Up	“Medium" Future Scale-Up	“High" Future Scale-Up
2012	400,000	400,000	600,000	800,000
2013	200,000	400,000	600,000	900,000
2014	200,000	400,000	600,000	900,000
2015	200,000	400,000	600,000	700,000
2016	200,000	400,000	600,000	600,000
Total	1,200,000	2,000,000	3,000,000	3,900,000

Number of adults (age 15 y and older) initiating ART between midpoint of the previous year and the midpoint of indicated year.

## Results

**Figure 1:**
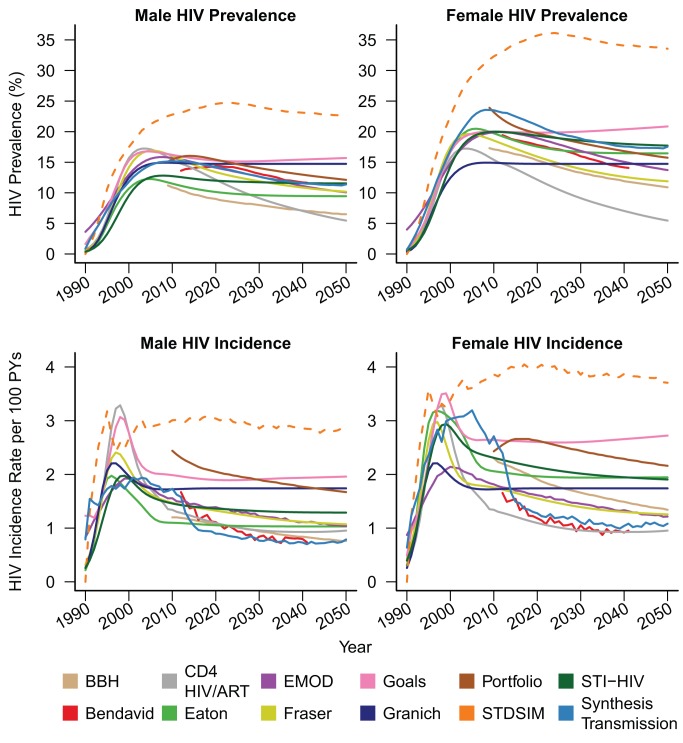
No-treatment counterfactual epidemic trends. Male (left) and female (right) HIV prevalence (top) and incidence (bottom) amongst 15- to 49-y-olds for counterfactual HIV epidemics with no ART. The STDSIM model is calibrated to a more severe epidemic in the Hlabisa subdistrict of KwaZulu-Natal Province, South Africa. The CD4 HIV/ART and Granich models do not stratify by sex, and the same prevalence and incidence curves are plotted for both sexes for these models. PYs, person-years.

Figure 1 shows HIV prevalence and HIV incidence in 15- to 49-y-old males and females simulated by each of the models under the counterfactual assumption of no ART provision.

Other epidemiologic statistics are presented in Table 3. The estimates of adult male HIV prevalence in year 2012 ranged between 10% and 16%, and estimates of female prevalence between 17% and 23%. Male HIV incidence in year 2012 ranged between 1.1 and 2.0 per 100 person-years, and female incidence ranged between 1.7 and 2.6. The STDSIM model calibrated to KwaZulu-Natal Province simulated a considerably larger burden of HIV, consistent with observation [47], with prevalences in year 2012 of 23% and 33% in males and females, respectively, and incidence rates of 3.0 and 3.9 per 100 person-years, respectively. All of the sex-stratified models simulated higher HIV prevalence in adult women than in men, with sex ratios in HIV prevalence in year 2012 between 1.2 and 1.7, and all of the models except for Bendavid simulated higher incidence in year 2012 in females than in males.

**Table 3:** Selected model outputs for counterfactual simulation with no ART.

Model Name	Age 15–49 y HIV Prevalence in Year 2012 (Percent)		Sex Ratio in Prevalence, Year 2012 (Female/Male)	Age 15–49 y HIV Incidence in Year 2012 (per 100 Person-Years)		Sex ratio in Incidence, Year 2012 (Female/Male)	Average Annual Population Growth Rate, Age 15+ y Population (per 100 People)		Year of Peak HIV Incidence		Percentage Change from Peak Incidence to year 2012 (Percent)		Percentage Change in Incidence, Year 2012 to 2020 (Percent)		Percentage Change in Incidence, Year 2020 to 2050 (Percent)	
	Male	Female		Male	Female		2012–2020	2012–2050	Male	Female	Male	Female	Male	Female	Male	Female
BBH	10.4	16.8	1.6	1.2	2.2	1.8	0.5	0.5					−9	−13	−30	−30
Bendavid	13.6	19.4	1.4	1.7	1.7	1.0	0.8	1.0^a^					−33	−29		
CD4 HIV/ART	14.8			1.3			1.5	1.1	1998		−61		−15		−13	
Eaton	11.1	19.2	1.7	1.1	2.0	1.9	1.4	1.5	1996	1997	−45	−36	−4	−4	−2	−1
EMOD	15.4	19.9	1.3	1.5	1.8	1.2	0.6	0.5	2001	2000	−23	−18	−7	−8	−25	−25
Fraser	15.4	18.0	1.2	1.4	1.7	1.2	−0.2	0.0	1997	1997	−40	−42	−8	−10	−19	−19
Goals	16.0	20.0	1.3	2.0	2.6	1.3	0.3	0.1	1998	1999	−36	−25	−3	−1	3	5
Granich	14.9			1.7			−0.2	−0.1	1997		−22		1		0	
HIV Portfolio	15.9	22.0	1.4	2.3	2.6	1.1	−0.8	−0.6					−11	3	−19	−18
STDSIM^b^	23.1	33.0	1.4	3.0	3.9	1.3	−1.3	−1.3	1995	2003	−6	3	2	2	−8	−7
STI-HIV	12.6	20.0	1.6	1.4	2.3	1.6	0.4	0.1	1999	1999	−28	−23	−5	−6	−6	−11
Synthesis Transmission	15.2	23.1	1.5	1.6	2.4	1.5	0.7	0.5	2001	2005	−18	−25	−43	−47	−12	−15

a Average annual growth rate for years 2012 to 2040.

b Model calibrated to HIV epidemic in KwaZulu-Natal Province.

Nearly all of the models projected declines in HIV incidence after 2012 in the absence of ART, but the magnitude of the projected natural changes between 2012 and 2050 varied widely from almost no change (Goals and Granich) to greater than 45% reduction (Bendavid and Synthesis Transmission).

Model projections of future national population growth in the absence of ART varied widely, ranging from a 6% reduction to a 13% increase in the population aged 15 y and older between the years 2012 and 2020. For comparison, the low and high variants for the projected total population growth from the United Nations Population Division over the same period (which incorporates some assumptions about ART provision) are 1.5% and 6.1% [48].

### Impact of ART on HIV Incidence

**Figure 2:**
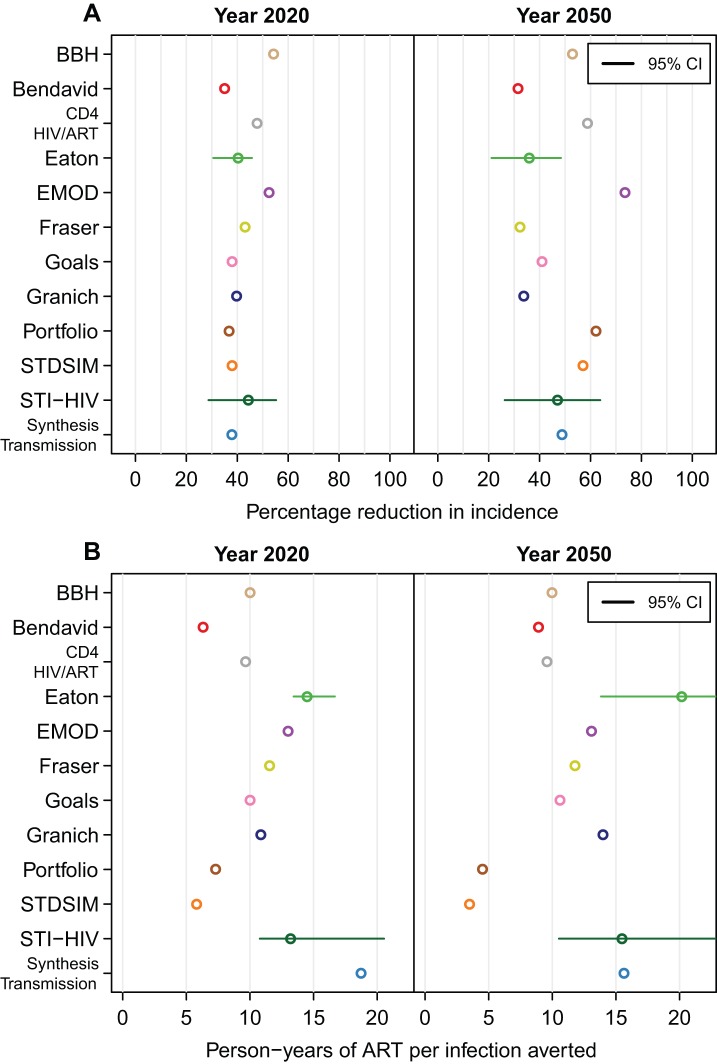
Impact of treatment for a scenario with eligibility at CD4≤350 cells/µl, 80% access, and 85% retention. (A) The percentage reduction in HIV incidence in the years 2020 and 2050 when eligibility for treatment is at CD4 count ≤350 cells/µl, 80% of individuals are treated, and 85% are retained on treatment after 3 y. (B) The cumulative number of person-years of ART provided per infection averted for the same scenario. Horizontal lines indicate 95% credible intervals (CI). For the Bendavid model, results in year 2040 are reported in the right panels.

Figure 2 presents the outcomes of an intervention starting in year 2012 with ART eligibility at CD4 count ≤350 cells/µl, reaching 80% of those requiring treatment, and retaining 85% of patients on ART after 3 y. This scenario reflects an optimised implementation of the current World Health Organization treatment guidelines [39] and the Joint United Nations Programme on HIV/AIDS definition for “universal access" of reaching 80% of those in need [32]. Compared to the no-treatment counterfactual scenario, ART provision reduced incidence in year 2020 by 35% to 54% across all models (Figure 2A). There was much greater variation, however, in the estimated long-term impact of the intervention. In year 2050, the range of the predicted reduction in incidence was from 32% to 74%. The relative impact of the ART intervention on HIV incidence decreased between 2020 and 2050 in four models and increased in seven.

### Number of Person-Years of ART per Infection Averted

There was considerable variation between models in estimates of the number of person-years of treatment per infection averted. For the scenario described above, the range of estimates for the number of person-years of ART per infection averted between 2012 and 2020 was between 6.3 and 18.7, and over the period 2012 to 2050, the range was 4.5 to 20.2 (Figure 2B). The four models with the greatest estimates of the number of person-years of ART provided per infection averted (Eaton, EMOD, STI-HIV Interaction, and Synthesis Transmission) all explicitly included variation in transmission by age (e.g., allowing for reduced impact through ART provision to older adults who are less sexually active and hence less likely to expose susceptible individuals), whereas the other models did not assume reduced transmission by older people. (STDSIM allows for decreased sexual activity for those older than 50 and has the lowest estimate of person-years of ART per infection averted, but simulates a much higher HIV incidence.)

### Determinants of Programme Impact

**Figure 3:**
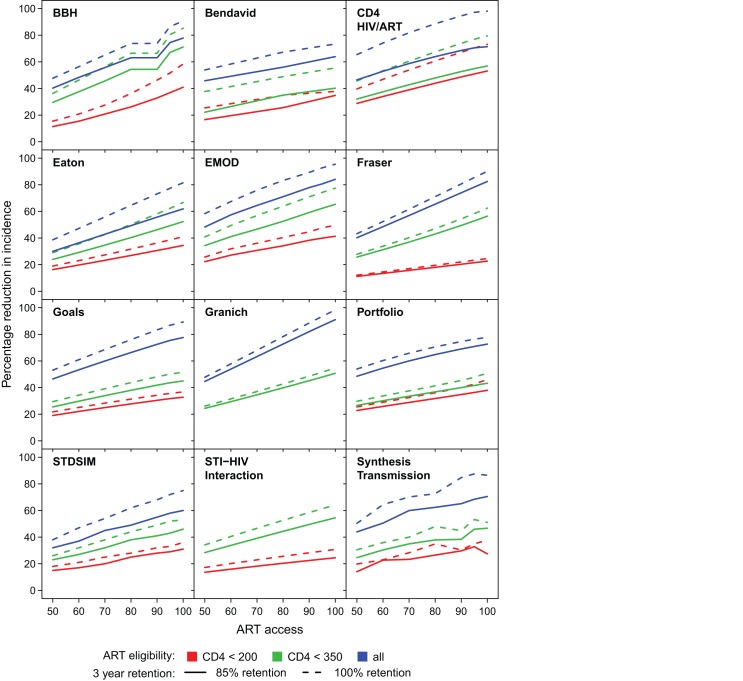
Proportion reduction in HIV incidence in year 2020. For each model, the proportion reduction in HIV incidence in year 2020 for increasing access levels from 50% to 100% (horizontal axis). ART eligibility thresholds are indicated by line colour; 85% retention is indicated by solid lines, and perfect 100% retention is indicated by dashed lines.

The impact on incidence of increasing access from 50% to 100%, improving 3-y programme retention from 85% to 100%, and changing the CD4 threshold for treatment eligibility, is shown for each model in Figure 3. The reduction in incidence increases approximately linearly with access in all models. In most models, improvements in retention in care led to greater impact of treatment on HIV incidence. The benefit of improving retention was minimal for the Fraser, Granich, and HIV Portfolio models. Each of these models regards individuals who have dropped out of treatment identically to untreated eligible individuals, allowing them to start treatment again on average within 1 y. In several models, improved retention means that the impact improves more rapidly with increasing access (i.e., the slope in reduction in incidence as access increases is steeper for higher retention).

**Figure 4:**
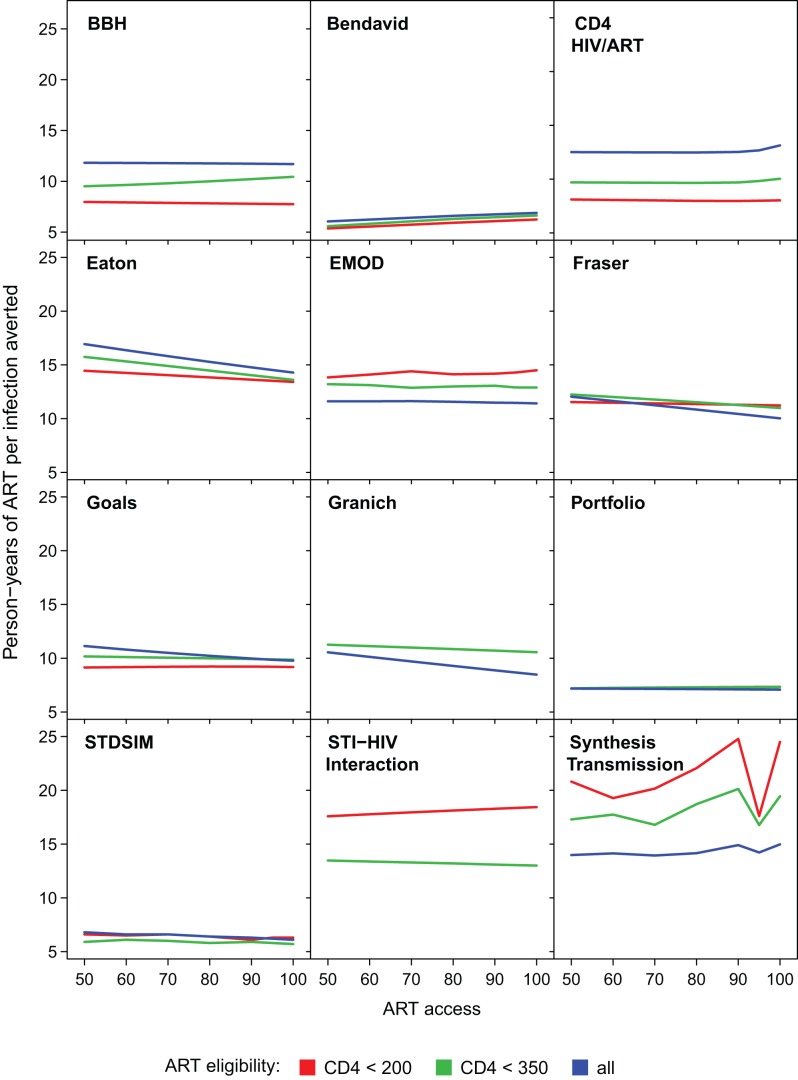
Cumulative number of person-years of ART provided per infection averted through year 2020. The cumulative person-years of ART provided per infection averted through the year 2020 for increasing access levels from 50% to 100% (horizontal axis), assuming 85% retention after 3 y. ART eligibility thresholds of are indicated by line colour. Varying retention did not affect trends between access and efficiency for any models.

Figure 4 shows how the number of person-years of ART provided per infection averted up to year 2020 varied in relation to the intervention programme. There were no consistent trends across all models. In some models, with earlier initiation of treatment, fewer years of ART were required per infection averted (efficiency increases), whereas the opposite was predicted in others. For all of the models except the Granich model, which does not include increased transmissibility during late-stage infection, it might be expected that treating at lower CD4 count would be more efficient, as it targets treatment towards individuals with the highest current infectiousness (as in the BBH, Bendavid, CD4 HIV/ART, Eaton, and Goals models). However this could be counteracted if stage of infection interacts with other processes such as decreased propensity to form new partnerships with ageing. For half of the models (BBH, Eaton, EMOD, Fraser, Goals, Granich, and HIV Portfolio), increasing the percentage of the population with access to treatment reduced the amount of treatment per infection averted, at least at earlier CD4 initiation thresholds. This increased efficiency is indicative of increasing returns due to “herd immunity" at high intervention coverage levels.

### Treatment Eligibility and the Theoretical Impact of “Test and Treat"

The models varied in their predictions as to the relative benefit of increasing treatment eligibility from a CD4 threshold of ≤200 cells/µl (national guidelines in some settings and close to actual experience in many) to ≤350 cells/µl (international guidelines) compared to further increasing eligibility to all infected individuals (Figure 3). The Bendavid, CD4 HIV/ART, Goals, HIV Portfolio, and Synthesis Transmission models all predicted that there would be only a relatively modest benefit in moving from initiation at ≤200 cells/µl to ≤350 cells/µl, and a much greater benefit in moving from initiation at ≤350 cells/µl to immediately upon diagnosis of HIV infection. In contrast, the BBH model simulated very little benefit in moving from the ≤350 cells/µl threshold to immediate eligibility. The Eaton, Fraser, and EMOD models showed similar benefits associated with each of the increments at moderate levels of access.

One important argument that has been made for immediate ART is that commitment of a large amount of ART now could reduce the cumulative amount of ART required in the future as a result of averted HIV infections [2],[49]. Whether such savings could occur was evaluated by investigating whether the cumulative person-years of treatment through year 2050 to implement immediate treatment is less than the amount of ART required when treating after the CD4 count falls below 350 cells/µl for the same levels of access and retention. In six (BBH, CD4 HIV/ART, Fraser, Goals, HIV Portfolio, and STDSIM) out of eleven models (excluding STI-HIV Interaction) this was not the case: increasing eligibility from CD4≤350 cells/µl to immediate initiation always required more person-years of treatment, even with “perfect" ART programmes (100% access and 100% retention). However, for the EMOD model, expanding eligibility from CD4≤350 cells/µl to all HIV-infected adults required fewer cumulative person-years of treatment in all intervention scenarios (including access as low as 60% and retention in care as low as 75%). The Synthesis Transmission model found expanding access to be ART-saving with 70% access and retention above 95%, or with 80% access and retention above 85%. The other three models that found that expanding access could be ART-saving required more demanding assumptions about programmes: according to the Granich model, immediate initiation would be ART-saving if access were above 90% and retention above 95%; according to the Eaton model, access and retention would need to exceed 95%; and according to the Bendavid model, access and retention would both need to be 100%.

In an intervention treating all HIV-infected adults with 95% access and 95% retention, three (CD4 HIV/ART, EMOD, and HIV Portfolio) out of nine models (excluding BBH, Bendavid, and STI-HIV Interaction) predicted that HIV incidence would fall below 0.1% per year by 2050. The Granich model, which was used to argue the case for HIV elimination using treatment, projected that incidence in South Africa would be 0.13% under this scenario, a 92% reduction (in the original published projections, there was an assumption that risk of infection would fall by an additional 40% due to other interventions [34]).

### Understanding Differences between Model Predictions

**Figure 5:**
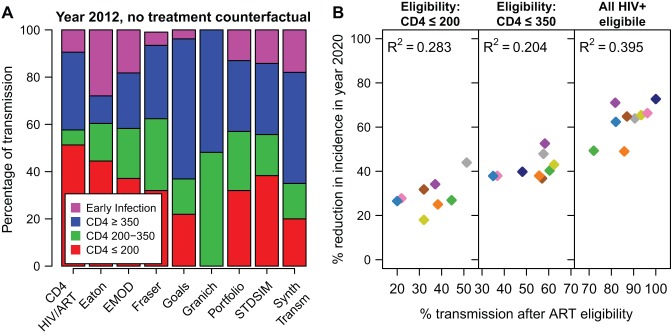
Impact of treatment by transmission in each CD4 category. (A) The percentage of all HIV transmissions from individuals in each CD4 cell count category in year 2012, in the no-ART counterfactual simulation. (B) The reduction in incidence in year 2020, for the 80% access and 85% retention scenario, according to the cumulative proportion of transmission that occurs after eligibility (A). For the scenario where all HIV-positive adults are eligible (“all HIV+ eligible"), the percentage of transmission after ART eligibility is the percentage of transmission that occurs after the end of primary HIV infection. Colours for models are the same as in Figures 1 and 2. The BBH, Bendavid, and STI-HIV Interaction models do not estimate the proportion of transmission in each CD4 category and are not included in this figure.

One factor expected to influence how much ART reduces HIV is the fraction of all transmission that occurs after individuals reach treatment eligibility thresholds, in the absence of any treatment [50]. Figure 5A shows the proportion of transmissions that occur from individuals in each CD4 count range in the counterfactual simulation in year 2012. Of the models that include a period of early infection, the percentage of new infections that occurs during this stage is between 4% and 28%, while between 20% and 51% of transmission results from individuals with CD4 cell count ≤200 cells/µl.

These percentages of transmission after ART eligibility can be compared with the percentage reduction in incidence in year 2020 (Figure 5B). Here, it is assumed that access is 80% and 3-y retention in care is 85%. Although this comparison explains why, within one model, earlier treatment initiation reduces HIV incidence more, the amount of between-model variation in projected impact explained by the distribution of transmission by CD4 count is modest. *R* ^2^ values for this relationship were 0.28, 0.20, and 0.40 for eligibility at CD4≤200, eligibility at CD4≤350, and immediate eligibility, respectively. The correlation did not improve when considering higher access or higher retention scenarios.

Two other factors hypothesized to explain the differences between the model projections are different assumptions about the efficacy of ART in reducing transmission—between 90% and 99%—and different assumptions about the outcomes of individuals who drop out from treatment programmes. To test the importance of these factors, selected intervention scenarios were repeated under the artificial assumption that an individual never transmits after initiating treatment (treatment is 100% efficacious at preventing transmission, and retention on treatment is 100%). This assumption increased the intervention impact in every model, but, surprisingly, did not reduce the variation in the results between models or improve the ability of factors such as different model assumptions about CD4 progression, HIV transmission, or the future trajectory of HIV incidence to explain the variation.

### Estimates of the Current Impact of ART in South Africa

**Figure 6:**
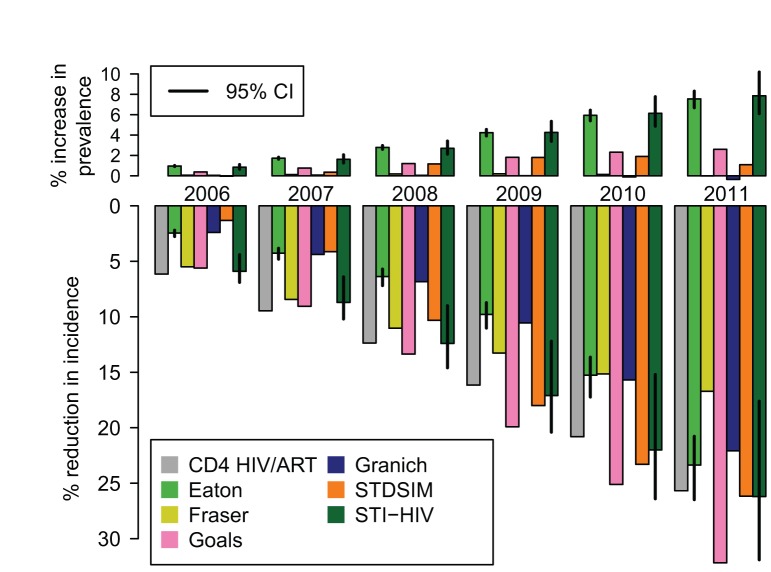
The impact of the existing ART programme in South Africa on HIV prevalence and incidence. The percentage increase in HIV prevalence (top) and the percentage reduction in HIV incidence rate (bottom) compared to what would have occurred in the absence of any ART for years the 2006 to 2011. These are estimated by comparing HIV prevalence and incidence in a model calibrated to the existing scale-up of ART in South Africa from 2001 to 2011 with a model simulation with no ART provision. The CD4 HIV/ART, Eaton, Goals, Granich, and STI-HIV Interaction models use the same estimates of the number starting ART each year (from [45]). Fraser uses an existing calibration to the ART scale-up in the Western Cape Province. STDSIM is calibrated to the number of people on ART in the Hlabisa subdistrict. Vertical lines on the Eaton and STI-HIV Interaction models indicate 95% credible intervals (CI).

Figure 6 shows the estimated impact of the current ART programme in South Africa on HIV prevalence and incidence. The CD4 HIV/ART, Eaton, Goals, Granich, and STI-HIV Interaction models used estimates of the number of adults starting treatment in South Africa in each year between 2001 and 2011 from [45], and the Fraser and STDSIM models used existing calibrations to ART coverage levels in the Western Cape and KwaZulu-Natal Provinces, respectively. All of the models predicted that ART should already have had a substantial impact on the HIV epidemic, estimating that HIV incidence in year 2011 was between 17% and 32% lower than it would have been in the absence of ART. The increasing impact on HIV incidence over time mirrors the steep increase every year in the number of people starting treatment during this period.

The impact on prevalence was more modest and less consistent across models. The Eaton and STI-HIV Interaction models estimated that prevalence is around 8% higher than it would have been without treatment (an absolute increase in prevalence of one percentage point) due to the increased survival for those infected with HIV. The Fraser and Granich models suggest that this effect is offset by the reductions in incidence, so that there is no net change in prevalence. It is unlikely that standard surveillance methods based on monitoring trends in prevalence would have detected this impact, despite the significant underlying reductions in incidence.

The estimated potential impact of further ART scale-up is summarised in Table 4. In the baseline scenario, where 400,000 people are started on ART each year, the models estimated that incidence would be reduced in 2016 by between 13% and 26% compared to the incidence rate in 2011. If 800,000 fewer people are put on ART, then between 39,000 and 186,000 more new adult HIV infections would occur over the period 2012 to 2016 than under the baseline scenario. If more people are put on ART—3.0 or 3.9 million over the next 5 y—then the models estimated that the number of new infections over the 5-y period would be reduced by 64,000 to 327,000 and 270,000 to 521,000, respectively, compared to the baseline. The table underscores that there are still substantial potential preventive benefits from expanding ART coverage in South Africa. The models that tended to estimate the greatest reduction in incidence in hypothetical programmes over the medium term (CD4 HIV/ART, Goals, and Granich) also tended to project greater reductions in incidence over the short term in these more realistic scenarios.

**Table 4:** HIV incidence rate per 100 person-years in year 2016 for different potential scenarios of future ART scale-up.

Model	“Low" Future Scale-Up		“Baseline" Future Scale-Up		“Medium" Future Scale-Up		“High" Future Scale-Up	
	HIV Incidence 2016^a^	Number Different from “Baseline"^b^	HIV Incidence 2016^a^	Number Different from “Baseline"^b^	HIV Incidence 2016^a^	Number Different from “Baseline"^b^	HIV Incidence 2016^a^	Number Different from “Baseline"^b^
Bendavid	1.20	39,000	1.14	—	1.06	−64,000	0.89	−270,000
CD4 HIV/ART	1.77	176,000	1.21	—	0.80	−327,000	0.54	−506,000
Eaton^c^	1.23 (1.07, 1.39)	106,000 (100,000, 113,000)	1.05 (0.89, 1.21)	—	0.79 (0.65, 0.95)	−111,000 (−118,000, −101,000)	0.70 (0.56, 0.83)	−310,000 (−334,000, −286,000)
Goals	1.50	186,000	1.16	—	0.88	−187,000	0.64	−419,000
Granich	1.34	142,000	1.14	—	0.87	−225,000	0.53	−521,000

Number of adults (age 15 y and older) initiating ART between midpoint of the previous year and the midpoint of indicated year.

a HIV incidence rate per 100 susceptible person-years amongst 15- to 49-y-olds at midpoint of year 2016.

b Cumulative number of additional new infections over the period mid-2011 to mid-2016 compared to “baseline" future scale-up scenario (rounded to nearest 1,000).

c The Eaton model reports posterior mean and 95% credible intervals.

## Discussion

The mathematical models used to simulate the impact of treatment on HIV incidence in South Africa are diverse in their structure, level of complexity, representation of the HIV transmission process and the ART intervention, and parameter choices. All twelve of the models compared in this analysis predicted that treatment could substantially reduce HIV incidence—even using past or existing treatment guideline eligibility criteria, provided that coverage is high. Only three (CD4 HIV/ART, EMOD, and HIV Portfolio) out of nine models (excluding BBH, Bendavid, and STI-HIV Interaction), however, predicted that treatment could reduce HIV incidence below 0.1% by year 2050 (the definition of “elimination" established by [34]), even with very high access and retention. When simulating the historical scale-up of ART in South Africa, the models indicated that ART may already have reduced HIV incidence by between 17% and 32% in 2011, compared to what would have been expected in the absence of ART.

Although there have been ad hoc informal model comparison exercises [51], collections of work using standardised assumptions for interventions [52], and thorough model comparisons involving a few research groups [53],[54], to our knowledge, this exercise is the first to bring together such a large number of independent modelling groups to examine the same set of interventions. We hope that this will provide a foundation for much more collaborative work.

In this study we set out to test whether different models of the potential impact of treatment on new HIV infections in South Africa would make similar predictions when implementing the same intervention scenarios. We found substantial consistency between the model projections of the impact of ART interventions on HIV incidence in the short term (8 y). However, there was more variation in the predicted longer term (38 y) reductions in incidence, and models also produced divergent estimates of the number of person-years of ART provided per infection averted. While establishing where models agree and disagree about the epidemiological impact of ART represents an important scientific finding in itself, the substantial variation in the long-term impact and efficiency of interventions demands further investigation and explanation.

Based on epidemiological theory and previous modelling studies, we hypothesized a number of model attributes that might explain differences in model predictions about the impact of ART, including the amount of transmission in different stages of HIV infection, the assumed efficacy of ART for preventing transmission, opportunities for treatment reinitiation following dropout from a treatment programme, the age and sex structure of the population, future population growth rates, the degree of heterogeneity and assortativity in sexual mixing, the future trajectory of HIV incidence in the absence of intervention, and the inclusion of changes in sexual behaviour over the past decade. There was indeed substantial variation between the models in their characterisation of each of these aspects of the system, largely reflecting the true uncertainties that persist even after decades of tremendous research into the epidemiology of HIV in South Africa. We were able to show that crude differences in the proportion of transmission at each stage of infection explained a modest amount of the variation in the short-term impact of ART, but less of the long-term impact. However, beyond this, findings from the models did not appear to clearly support any of these hypotheses in univariate analyses, likely because of the large number of processes that interact nonlinearly to create HIV epidemics and interventions. For example, projecting a seemingly simple quantity such as the number of person-years of ART that will be provided in an intervention depends on future population growth, the natural trend in the epidemic, the proportion of HIV-infected individuals qualifying for treatment, retention and survival on ART, and the impact that ART provision has on future HIV incidence. This situation contrasts with that of an earlier exercise that compared predictions of the impact of male circumcision interventions [51], where the relationship between the established efficacy of the intervention and population-level impact was less complicated.

Having investigated the extent to which differences in ART programmes determine differences in results, the natural focus for future model comparison studies should be to explore the contribution of other hypotheses through incrementally standardising biological, behavioural, and demographic model parameters, and calibrating models to the same levels of HIV prevalence and incidence. A systematic approach to standardising model parameters would identify which parameters most significantly influence the results and guide priorities for future data collection. The HIV Modelling Consortium (http://www.hivmodelling.org) will coordinate such research efforts in coming months to investigate the extent to which variation in model predictions is driven by differences in underlying models of sexual mixing, or different models of the natural history of infection and epidemic trajectory.

Although our experiment and analysis has focused on how factors included in models can affect model predictions, it is important to note that if all models exclude an important aspect of the system, they could all be wrong. Early models of the impact of ART on HIV incidence were very focused on the concern that increased sexual risk behaviour might offset the reduction in transmission for those on treatment, but for this exercise all of the models assumed that population risk behaviour would not change in response to the introduction of ART. This may be a reasonable assumption given consistent evidence that patients report safer sexual behaviour after starting ART [55]–[59] and given the relative lack of information from sub-Saharan Africa about how the untreated and HIV-negative population responds to the availability of treatment [60]. But in other epidemic settings the availability of ART has been associated with receding gains in protective behaviour [61]–[63], and monitoring this in sub-Saharan African settings will be a priority for surveillance over coming years. The models also all assumed high efficacy of ART to reduce transmission. True effectiveness will depend on adherence and the level of viral suppression, which is mainly determined by adherence levels. While there are some data from South Africa on viral suppression rates outside carefully controlled trial settings [64], further information on this and on patterns of acquired and transmitted resistance will help in the calibration of models. Only one of the models in this exercise (Synthesis Transmission) explicitly incorporated the effect of antiretroviral drug resistance on the impact of ART interventions. Models have predicted that antiretroviral drug resistance could be widespread in sub-Saharan Africa in coming decades [20], which could eventually lead to the spread of transmitted drug resistance [65],[66]. This could affect the long-term costs and efficacy of treatment-as-prevention strategies [67].

Another finding from systematically comparing models is that often seemingly independent modelling studies rely on the same limited data. Nearly all of the models relied on two sources to derive parameters for elevated infectiousness during the first few weeks of infection [68],[69], but both of these sources are based principally on data from a few retrospective couples in Rakai, Uganda [70] (see [71]). This highlights both how invaluable these data are and also the importance of recognising the dependencies between seemingly independent modelling studies. However, even using the same data, models may reach different conclusions. The Eaton, EMOD, and Fraser models all in some way used the estimates of early HIV infectivity from [69] but estimate very different contributions of this stage to overall HIV transmission (Figure 4A), and the three models all reached different conclusions from those in another recent modelling study relying on these same estimates [72].

The purpose of this exercise was not to draw conclusions or recommendations about specific ART intervention strategies, but rather to test the hypothesis that a range of different models would come to similar conclusions about the impact of ART on HIV incidence when the same interventions were modelled. The simulated interventions were artificially simple and stylized to enable comparison between models. These did not explicitly simulate the steps of HIV testing, diagnosis, linkage to care, and adherence to ART required to achieve the access levels specified in the intervention scenarios (although several of the models include facility for this and have investigated this in independent analyses). Interpretation of models simulating high levels of treatment coverage should be cautioned by data suggesting that at present fewer than one-third of patients in sub-Saharan Africa are continuously retained in care from HIV diagnosis to ART initiation [73], and that barriers remain to access to and uptake of HIV testing [32]. The models assumed that all individuals eligible for treatment were equally likely to access treatment, which might not be true in practice (for example, women are more likely to start treatment than men [74]). The comparison scenario (counterfactual) against which interventions were evaluated assumed no treatment at all, which made it easier to compare models, but is clearly not the relevant benchmark for policy-makers. This study has also considered treatment in isolation from other interventions, even as there is broad consensus that “combination prevention" strategies are presently the best strategy for attacking the epidemic [41],[75].

We hope that this study will help to characterise the models that are being used to investigate questions related to the impact of HIV treatment and enable those who rely on models for decision-making to think critically about how the assumptions underlying models affect the results. The relative consistency between models' estimates of the short-term epidemiological impact of ART, including the impact of the existing ART programme, provides some reassurance that model projections on this time scale may be relatively robust to the substantial uncertainties in parameters and systems. This is a significant result considering that such short-term projections are often the most relevant for policy and resource allocation questions. On the other hand, the substantial variation in long-term epidemiological impacts and efficiency of ART, upon which arguments of substantial epidemic reduction and cost savings hinge, suggests that results in these areas from any single model should be extrapolated with caution. Care should be taken to ensure that models evaluating the long-term costs, benefits, and cost-effectiveness of treatment programmes adequately communicate the degree and myriad sources of uncertainty that influence these outputs.

A common question when faced with a diversity of model results is whether some models are “better" or “worse". Without data against which to test the predictions of models, it is not possible to answer this question in a study such as this, nor is this the correct question to be asking. Rather, users of model outputs should ask whether models include the necessary components to capably answer the specific questions at hand, and whether the models make credible assumptions in light of the information available, and choose models accordingly. Evaluated along these guidelines, the most appropriate models will vary between applications, so there is no single “best" model. However, in this exercise, the models that tended to project more “pessimistic" outcomes for the interventions seemed to do so for important reasons. For example, models that estimated poorer efficiency of ART for averting infections tended to be those that simulated ART provision for those at older ages, who might be at lower risk of transmitting, or included the elevated risk of transmission for those failing treatment, whereas models with more optimistic predictions assumed that risk behaviour did not vary by age or that transmission was fully suppressed immediately upon beginning treatment until death on ART or dropout. Artificial convergence of models should be avoided when true uncertainties persist about the system. It is incumbent upon modellers to incorporate and communicate uncertainty in projections, and identify which components of the system account for the uncertainty. For this exercise, only one model (STI-HIV Interaction) included a comprehensive analysis accounting for uncertainty about basic epidemiology and intervention efficacy. While the focus of the study was on variation between models, it is interesting to observe that the 95% credible interval representing parameter uncertainty for this model encompassed the point estimates of the other eleven models.

Fortunately there will be important new opportunities in the near future to test, validate, and improve epidemiological models of HIV treatment. These include comparing projections to the experience of expanded ART in industrialised countries [61],[63], the observed impact of ART in well-characterised communities [76], and results of a number of community-randomized trials of treatment as prevention that will soon be underway [44]. As new data are reported, the accuracy of models projecting the impact of treatment as prevention should improve, and we expect that validated and scientifically based model projections will continue to be central in understanding how ART can have the greatest impact in mitigating the global HIV epidemic.

## Abbreviations

ART: antiretroviral therapy

## References

[1] Montaner JSG, Hogg R, Wood E, Kerr T, Tyndall M (2006). The case for expanding access to highly active antiretroviral therapy to curb the growth of the HIV epidemic.. Lancet.

[2] De Cock KM, Gilks CF, Lo Y-R, Guerma T (2009). Can antiretroviral therapy eliminate HIV transmission?. Lancet.

[3] Garnett GP, Baggaley RF (2009). Treating our way out of the HIV pandemic: could we, would we, should we?. Lancet.

[4] Cohen J (2011). Halting HIV/AIDS epidemics.. Science.

[5] Granich R, Gupta S, Suthar A, Smyth C, Hoos D (2011). Antiretroviral therapy in prevention of HIV and TB: update on current research efforts.. Curr HIV Res.

[6] Quinn TC, Wawer MJ, Sewankambo N, Serwadda D, Li C (2000). Viral load and heterosexual transmission of human immunodeficiency virus type 1. Rakai Project Study Group.. N Engl J Med.

[7] Fraser C, Hollingsworth TD, Chapman R, de Wolf F, Hanage WP (2007). Variation in HIV-1 set-point viral load: epidemiological analysis and an evolutionary hypothesis.. Proc Natl Acad Sci U S A.

[8] Vernazza PL, Troiani L, Flepp MJ, Cone RW, Schock J (2000). Potent antiretroviral treatment of HIV-infection results in suppression of the seminal shedding of HIV. The Swiss HIV Cohort Study.. AIDS.

[9] Cu-Uvin S, Caliendo AM, Reinert S, Chang A, Juliano-Remollino C (2000). Effect of highly active antiretroviral therapy on cervicovaginal HIV-1 RNA.. AIDS.

[10] Baggaley RF, Ferguson NM, Garnett GP (2005). The epidemiological impact of antiretroviral use predicted by mathematical models: a review.. Emerg Themes Epidemiol.

[11] Dodd PJ, Garnett GP, Hallett TB (2010). Examining the promise of HIV elimination by “test and treat" in hyperendemic settings.. AIDS.

[12] Anderson RM, Gupta S, May RM (1991). Potential of community-wide chemotherapy or immunotherapy to control the spread of HIV-1.. Nature.

[13] Gilliam BL, Dyer JR, Fiscus SA, Marcus C, Zhou S (1997). Effects of reverse transcriptase inhibitor therapy on the HIV-1 viral burden in semen.. J Acquir Immune Defic Syndr Hum Retrovirol.

[14] Blower SM, Gershengorn HB, Grant RM (2000). A tale of two futures: HIV and antiretroviral therapy in San Francisco.. Science.

[15] Dangerfield BC, Fang Y, Roberts CA (2001). Model-based scenarios for the epidemiology of HIV/AIDS: the consequences of highly active antiretroviral therapy.. Syst Dyn Rev.

[16] Law MG, Prestage G, Grulich A, Van de Ven P, Kippax S (2001). Modelling the effect of combination antiretroviral treatments on HIV incidence.. AIDS.

[17] Velasco-Hernandez JX, Gershengorn HB, Blower SM (2002). Could widespread use of combination antiretroviral therapy eradicate HIV epidemics?. Lancet Infect Dis.

[18] Xiridou M, Geskus R, De Wit J, Coutinho R, Kretzschmar M (2003). The contribution of steady and casual partnerships to the incidence of HIV infection among homosexual men in Amsterdam.. AIDS.

[19] Gray RH, Li X, Wawer MJ, Gange SJ, Serwadda D (2003). Stochastic simulation of the impact of antiretroviral therapy and HIV vaccines on HIV transmission; Rakai, Uganda.. AIDS.

[20] Blower S, Bodine E, Kahn J, McFarland W (2005). The antiretroviral rollout and drug-resistant HIV in Africa: insights from empirical data and theoretical models.. AIDS.

[21] Baggaley RF, Garnett GP, Ferguson NM (2006). Modelling the impact of antiretroviral use in resource-poor settings.. PLoS Med.

[22] McCormick AW, Walensky RP, Lipsitch M, Losina E, Hsu H (2007). The effect of antiretroviral therapy on secondary transmission of HIV among men who have sex with men.. Clin Infect Dis.

[23] Salomon JA, Hogan DR (2008). Evaluating the impact of antiretroviral therapy on HIV transmission.. AIDS.

[24] Attia S, Egger M, Müller M, Zwahlen M, Low N (2009). Sexual transmission of HIV according to viral load and antiretroviral therapy: systematic review and meta-analysis.. AIDS.

[25] Reynolds SJ, Makumbi F, Nakigozi G, Kagaayi J, Gray RH (2011). HIV-1 transmission among HIV-1 discordant couples before and after the introduction of antiretroviral therapy.. AIDS.

[26] Donnell D, Baeten JM, Kiarie J, Thomas KK, Stevens W (2010). Heterosexual HIV-1 transmission after initiation of antiretroviral therapy: a prospective cohort analysis.. Lancet.

[27] Glynn JR, Price A, Floyd S, Molesworth A, Kayuni N (2011). Anitretroviral therapy reduces HIV transmission in discordant couples in northern Malawi.. Sex Transm Infect.

[28] Cohen MS, Chen YQ, McCauley M, Gamble T, Hosseinipour MC (2011). Prevention of HIV-1 infection with early antiretroviral therapy.. N Engl J Med.

[29] Lodwick R, Costagliola D, Reiss P, Torti C, Teira R (2010). Triple-class virologic failure in HIV-infected patients undergoing antiretroviral therapy for up to 10 years.. Arch Intern Med.

[30] Boulle A, Van Cutsem G, Hilderbrand K, Cragg C, Abrahams M (2010). Seven-year experience of a primary care antiretroviral treatment programme in Khayelitsha, South Africa.. AIDS.

[31] Nash D, Katyal M, Brinkhof MWG, Keiser O, May M (2008). Long-term immunologic response to antiretroviral therapy in low-income countries: a collaborative analysis of prospective studies.. AIDS.

[32] Joint United Nations Programme on HIV/AIDS (2010). Towards universal access: scaling up priority HIV/AIDS interventions in the health sector.. http://whqlibdoc.who.int/publications/2010/9789241500395_eng.pdf.

[33] Joint United Nations Programme on HIV/AIDS (2011). World AIDS Day Report 2011. How to get to zero: faster, smarter, better.. http://www.unaids.org/en/media/unaids/contentassets/documents/unaidspublication/2011/JC2216_WorldAIDSday_report_2011_en.pdf.

[34] Granich RM, Gilks CF, Dye C, De Cock KM, Williams BG (2009). Universal voluntary HIV testing with immediate antiretroviral therapy as a strategy for elimination of HIV transmission: a mathematical model.. Lancet.

[35] Wagner BG, Blower S (2009). Voluntary universal testing and treatment is unlikely to lead to HIV elimination: a modeling analysis.. http://dx.doi.org/10.1038/npre.2009.3917.1.

[36] Kretzschmar ME, van der Loeff MF, Coutinho RA (2012). Elimination of HIV by test and treat.. AIDS.

[37] Bacaër N, Pretorius C, Auvert B (2010). An age-structured model for the potential impact of generalized access to antiretrovirals on the South African HIV epidemic.. Bull Math Biol.

[38] Bendavid E, Brandeau ML, Wood R, Owens DK (2010). Comparative effectiveness of HIV testing and treatment in highly endemic regions.. Arch Intern Med.

[39] World Health Organization (2010). Antiretroviral therapy for HIV infection in adults and adolescents: recommendations for a public health approach, 2010 revision.. http://whqlibdoc.who.int/publications/2010/9789241599764_eng.pdf.

[40] PEPFAR Scientific Advisory Board (2011). PEPFAR Scientific Advisory Board recommendations for the Office of the US Global AIDS Coordinator: implications of HPTN 052 for PEPFAR's treatment programs.. http://www.pepfar.gov/documents/organization/177126.pdf.

[41] Schwartländer B, Stover J, Hallett T, Atun R, Avila C (2011). Towards an improved investment approach for an effective response to HIV/AIDS.. Lancet.

[42] Hontelez JA, de Vlas SJ, Tanser F, Bakker R, Bärnighausen T (2011). The impact of the new WHO antiretroviral treatment guidelines on HIV epidemic dynamics and cost in South Africa.. PLoS ONE.

[43] Johnson LF, Hallett TB, Rehle TM, Dorrington RE (2012). The effect of changes in condom usage and antiretroviral treatment coverage on human immunodeficiency virus incidence in South Africa: a model-based analysis.. J R Soc Interface.

[44] Boily MC, Mâsse B, Alsallaq R, Padian NS, Eaton JW (2012). HIV treatment as prevention: considerations in the design, conduct, and analysis of cluster randomized controlled trials of combination HIV prevention.. PLoS Med.

[45] Johnson LF (2012). Access to antiretroviral treatment in South Africa, 2004–2011.. South Afr J HIV Med.

[46] South Africa National AIDS Council (2011). National strategic plan on HIV, STIs and TB: 2012–2016 summary.. http://www.doh.gov.za/docs/stratdocs/2012/NSPsum.pdf.

[47] Shisana O, Rehle T, Simbayi LC, Zuma K, Jooste S (2009). South African national HIV prevalence, incidence, behaviour and communication survey 2008: a turning tide amongst teenagers?.

[48] Population Division of the Department of Economic and Social Affairs of the United Nations Secretariat (2011). World population prospects: the 2010 revision.. http://esa.un.org/unpd/wpp/index.htm.

[49] The HIV Modelling Consortium Treatment as Prevention Editorial Writing Group (2012). HIV treatment as prevention: models, data, and questions—Towards evidence-based decision-making.. PLoS Med.

[50] Anderson RM, May RM (1992). Infectious diseases of humans: dynamics and control.

[51] UNAIDS/WHO/SACEMA Expert Group on Modelling the Impact and Cost of Male Circumcision for HIV Prevention (2009). Male circumcision for HIV prevention in high HIV prevalence settings: what can mathematical modelling contribute to informed decision making?. PLoS Med.

[52] Hankins CA, Glasser JW, Chen RT (2011). Modeling the impact of RV144-like vaccines on HIV transmission.. Vaccine.

[53] Halloran ME, Ferguson NM, Eubank S, Longini IM, Cummings DA (2008). Modeling targeted layered containment of an influenza pandemic in the United States.. Proc Natl Acad Sci U S A.

[54] Althaus CL, Turner KM, Schmid BV, Heijne JC, Kretzschmar M (2011). Transmission of Chlamydia trachomatis through sexual partnerships: a comparison between three individual-based models and empirical data.. J R Soc Interface.

[55] Bateganya M, Colfax G, Shafer LA, Kityo C, Mugyenyi P (2005). Antiretroviral therapy and sexual behavior: a comparative study between antiretroviral-naive and -experienced patients at an urban HIV/AIDS care and research center in Kampala, Uganda.. AIDS Patient Care STDS.

[56] Bunnell R, Ekwaru JP, Solberg P, Wamai N, Bikaako-Kajura W (2006). Changes in sexual behavior and risk of HIV transmission after antiretroviral therapy and prevention interventions in rural Uganda.. AIDS.

[57] Eisele TP, Mathews C, Chopra M, Lurie MN, Brown L (2009). Changes in risk behavior among HIV-positive patients during their first year of antiretroviral therapy in Cape Town South Africa.. AIDS Behav.

[58] McClelland RS, Graham SM, Richardson BA, Peshu N, Masese LN (2010). Treatment with antiretroviral therapy is not associated with increased sexual risk behavior in Kenyan female sex workers.. AIDS.

[59] Venkatesh KK, de Bruyn G, Lurie MN, Mohapi L, Pronyk P (2010). Decreased sexual risk behavior in the era of HAART among HIV-infected urban and rural South Africans attending primary care clinics.. AIDS.

[60] Venkatesh KK, Flanigan TP, Mayer KH (2011). Is expanded HIV treatment preventing new infections? Impact of antiretroviral therapy on sexual risk behaviors in the developing world.. AIDS.

[61] Bezemer D, de Wolf F, Boerlijst MC, van Sighem A, Hollingsworth TD (2008). A resurgent HIV-1 epidemic among men who have sex with men in the era of potent antiretroviral therapy.. AIDS.

[62] Smith MK, Powers KA, Muessig KE, Miller WC, Cohen MS (2012). HIV treatment as prevention: the utility and limitations of ecological observation.. PLoS Med.

[63] Wilson DP (2012). HIV treatment as prevention: natural experiments highlight limits of antiretroviral treatment as HIV prevention.. PLoS Med.

[64] Barth RE, van der Loeff MF, Schuurman R, Hoepelman AI, Wensing AM (2010). Virological follow-up of adult patients in antiretroviral treatment programmes in sub-Saharan Africa: a systematic review.. Lancet Infect Dis.

[65] Vardavas R, Blower S (2007). The emergence of HIV transmitted resistance in Botswana: “when will the WHO detection threshold be exceeded?". PLoS ONE.

[66] Phillips AN, Pillay D, Garnett G, Bennett D, Vitoria M (2011). Effect on transmission of HIV-1 resistance of timing of implementation of viral load monitoring to determine switches from first to second-line antiretroviral regimens in resource-limited settings.. AIDS.

[67] Wagner BG, Kahn JS, Blower S (2010). Should we try to eliminate HIV epidemics by using a “Test and Treat" strategy?. AIDS.

[68] Boily M-C, Baggaley RF, Wang L, Masse B, White RG (2009). Heterosexual risk of HIV-1 infection per sexual act: systematic review and meta-analysis of observational studies.. Lancet Infect Dis.

[69] Hollingsworth TD, Anderson RM, Fraser C (2008). HIV-1 transmission, by stage of infection.. J Infect Dis.

[70] Wawer MJ, Gray RH, Sewankambo NK, Serwadda D, Li X (2005). Rates of HIV-1 transmission per coital act, by stage of HIV-1 infection, in Rakai, Uganda.. J Infect Dis.

[71] Cohen MS, Dye C, Fraser C, Miller WC, Powers KA (2012). HIV treatment as prevention: debate and commentary—will early infection compromise treatment-as-prevention strategies?. PLoS Med.

[72] Powers KA, Ghani AC, Miller WC, Hoffman IF, Pettifor AE (2011). The role of acute and early HIV infection in the spread of HIV and implications for transmission prevention strategies in Lilongwe, Malawi: a modelling study.. Lancet.

[73] Rosen S, Fox MP (2011). Retention in HIV care between testing and treatment in sub-Saharan Africa: a systematic review.. PLoS Med.

[74] Cornell M, Grimsrud A, Fairall L, Fox MP, van Cutsem G (2010). Temporal changes in programme outcomes among adult patients initiating antiretroviral therapy across South Africa, 2002–2007.. AIDS.

[75] The US President's Emergency Plan for AIDS Relief (2011 August). Guidance for the prevention of sexually transmitted HIV infections.. http://www.pepfar.gov/documents/organization/171303.pdf.

[76] Maher D, Biraro S, Hosegood V, Isingo R, Lutalo T (2010). Translating global health research aims into action: the example of the ALPHA network.. Trop Med Int Health.

[77] Stover J, Bollinger L, Avila C (2011). Estimating the impact and cost of the WHO 2010 recommendations for antiretroviral therapy.. AIDS Res Treat.

[78] Stover J, Johnson P, Hallett T, Marston M, Becquet R (2010). The Spectrum projection package: improvements in estimating incidence by age and sex, mother-to-child transmission, HIV progression in children and double orphans.. Sex Transm Infect.

[79] Tanser F, Hosegood V, Bärnighausen T, Herbst K, Nyirenda M (2008). Cohort profile: Africa Centre Demographic Information System (ACDIS) and population-based HIV survey.. Int J Epidemiol.

[80] AIDS 2031 Commission (2010). AIDS in an uncertain world: the report of the AIDS 2031 Commission.

[81] Long E, Brandeau M (2010). The cost-effectiveness and population outcomes of expanded HIV screening and antiretroviral treatment in the United States.. Ann Intern Med.

[82] Hontelez JA, Lurie MN, Newell ML, Bakker R, Tanser F (2011). Ageing with HIV in south africa.. AIDS.

[83] Hontelez JA, Nagelkerke N, Bärnighausen T, Bakker R, Tanser F (2011). The potential impact of RV144-like vaccines in rural South Africa: a study using the STDSIM microsimulation model.. Vaccine.

[84] Johnson L, Dorrington R, Bradshaw D, Pillay-Van Wyk V, Rehle T (2009). Sexual behaviour patterns in South Africa and their association with the spread of HIV: insights from a mathematical model.. Demogr Res.

